# Novel CRISPR/Cas applications in plants: from prime editing to chromosome engineering

**DOI:** 10.1007/s11248-021-00238-x

**Published:** 2021-03-01

**Authors:** Teng-Kuei Huang, Holger Puchta

**Affiliations:** Botanical Institute II, Karlsruhe Institute of Technology, POB 6980, 76049 Karlsruhe, Germany

**Keywords:** CRISPR, Cas9, Cas12a, Gene editing, Chromosome engineering

## Abstract

In the last years, tremendous progress has been made in the development of CRISPR/Cas-mediated genome editing tools. A number of natural CRISPR/Cas nuclease variants have been characterized. Engineered Cas proteins have been developed to minimize PAM restrictions, off-side effects and temperature sensitivity. Both kinds of enzymes have, by now, been applied widely and efficiently in many plant species to generate either single or multiple mutations at the desired loci by multiplexing. In addition to DSB-induced mutagenesis, specifically designed CRISPR/Cas systems allow more precise gene editing, resulting not only in random mutations but also in predefined changes. Applications in plants include gene targeting by homologous recombination, base editing and, more recently, prime editing. We will evaluate these different technologies for their prospects and practical applicability in plants. In addition, we will discuss a novel application of the Cas9 nuclease in plants, enabling the induction of heritable chromosomal rearrangements, such as inversions and translocations. This technique will make it possible to change genetic linkages in a programmed way and add another level of genome engineering to the toolbox of plant breeding. Also, strategies for tissue culture free genome editing were developed, which might be helpful to overcome the transformation bottlenecks in many crops. All in all, the recent advances of CRISPR/Cas technology will help agriculture to address the challenges of the twenty-first century related to global warming, pollution and the resulting food shortage.

## Significance statement

Genome editing tools are evolving rapidly. They enable the generation of single or multiple mutations at the desired loci of the plant genome and thus the targeted removal of undesirable or desirable insertion of beneficial traits in crop plants. This review evaluates various CRISPR/Cas-mediated technologies, including recent applications of prime editing and heritable chromosomal rearrangements, in terms of prospects and applicability. The article thus provides the reader not only with an overview of the latest developments in plant genome editing technologies, but also with decision-making aids for the targeted use of these tools for specific fundamental science questions or applications.

**Stefan Schillberg**, *Fraunhofer Institute for Molecular Biology and Applied Ecology IME, Forckenbeckstrasse 6, 52,074 Aachen, Germany.*

**Diego Orzaez**, *Instituto de Biología Molecular y Celular de Plantas (IBMCP), Universitat Politècnica de València-Consejo Superior de Investigaciones Científicas (CSIC), Valencia, Spain*.

## Introduction

**Fig. 1:**
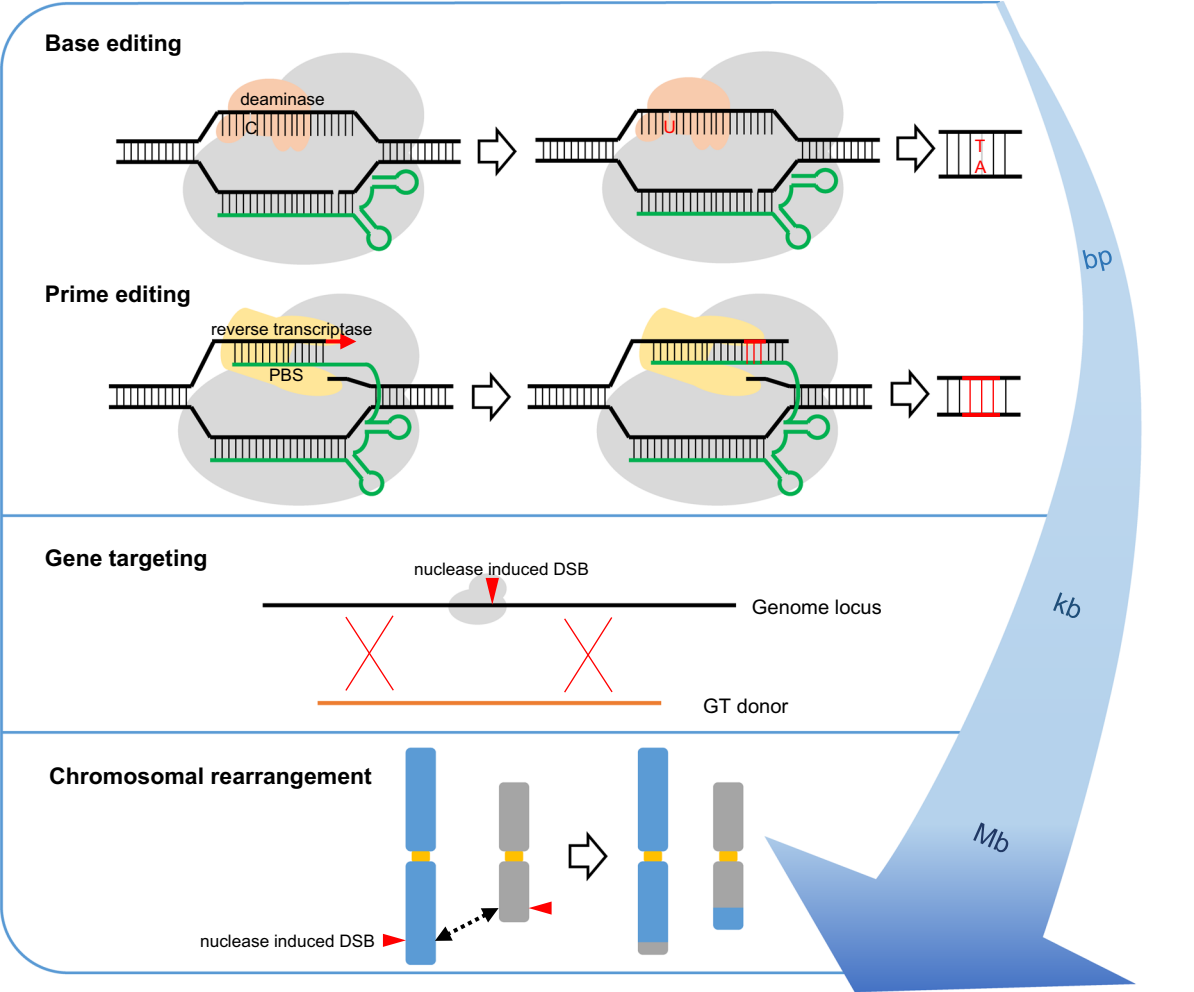
Tools for precise plant genome modification. Using CRISPR/Cas, it is now possible to induce changes in plant genomes from a single nucleotide to the restructuring of whole chromosomes on the Mb scale

In the middle of the last century, for the first time, plant breeders used the artificial induction of mutations to obtain new varieties. This was achieved by applying genotoxic agents, such as ionizing radiation, which randomly induce multiple genomic double-strand breaks (DSBs) (Stadler 1928). In plants, non-homologous end joining (NHEJ) is the dominant pathway of DSB repair, which often results in mutations at the break site (Puchta 2005). After it had become possible to use site-specific nucleases (SSNs) in multicellular eukaryotes (Puchta et al. 1993), the enzymatic induction of single genomic DSBs came into reach. Different kinds of artificial nucleases, such as Zinc-finger nucleases (ZFNs) and Transcription activator-like effector nucleases (TALENs), have been developed to target DSBs to preselected, unique positions in the genome (Voytas 2013). In principle, SSNs can be used most efficiently for mutagenesis by inducing error-prone NHEJ DSB repair in plants (Salomon and Puchta 1998). At the same time, they can also be used to increase homologous recombination (HR)-mediated gene targeting (GT) by several orders of magnitude (Puchta et al. 1996). In most cases, especially in basic science, reverse genetics approaches aim to generate null mutants to study the function of genes. However, like many other mutations that cause phenotypic changes, beneficial traits in agriculture are often due to gain or change of function. Therefore, the establishment of molecular tools for precise gene modifications is required in agriculture. Although many editing tools have been shown to work efficiently in other organisms, they might be of limited applicability in plants due to cellular or environmental differences. Thus, these tools often have not only to be tested but also adapted before they can be used in plants. Over the last few years, CRISPR/Cas-derived genome engineering technologies—due to their huge potential for medicine and all fields of biology and biotechnology—have been developed extremely fast. The number of approaches that are worth evaluating for their potential application in plants rises continuously. Besides various improvements in base editing (BE), a brand-new design for precise genome editing, prime editing (PE), has been developed. Moreover, CRISPR/Cas induced large chromosomal rearrangement (CR) have been achieved recently, making the breakage as well as the formation of genetic linkages an option for application in crops. As a number of excellent reviews has been published recently on different aspects of CRISPR/Cas applications in plants (Atkins and Voytas 2020; Chen et al. 2019; Schindele et al. 2020; Zhang et al. 2019; Zhu et al. 2020), our review will mainly focus on genome modification tools derived from CRISPR/Cas that were developed in the last two years and successfully applied in plants—from single bases to Mb changes (Fig. 1). We will discuss the potential, but also the limitations, of the respective approaches.

### Development of engineered CRISPR/Cas for genome editing

The broadest application of the CRISPR/Cas system in plants is the locus-specific DSB induction into genomic DNA to achieve mutagenesis. There are three CRISPR/Cas variants that are most widely used in plants: *Streptococcus pyogenes* Cas9 (SpCas9) and *Staphylococcus aureus* (SaCas9) from the type II system; and *Lachnospiraceae bacterium* ND2006 (LbCas12a), also called LbCpf1 from the type V system (Jinek et al. 2012; Ran et al. 2015; Zetsche et al. 2015). The use of different orthologs of CRISPR/Cas provides many benefits, such as the expansion of available target sites via different protospacer adjacent motifs (PAMs), the generation of different sizes of insertion-deletion mutations (Indels) or multiplex editing using different nucleases. Moreover, different enzyme activities might be targeted by Cas-mediated DNA binding for more complex manipulation of transcriptional or epigenetic state (Puchta 2016). LbCas12a differs in some intriguing properties from Cas9. Its TTTV PAM can be used to target AT-rich genomic regions. Moreover, LbCas12a frequently causes larger deletions than Cas9 due to its protruding single-strand ends after cleavage. Protein engineering of the Cas proteins further broadens the range of applications. By inducing inactivating mutations in both, the HNH domain and the RuvC domain, dCas9, a protein without nuclease activity but with DNA-binding activity can be obtained. On the other hand, inactivation of only one of the nuclease domains results in nCas9, a protein with nickase activity (Barrangou and Marraffini 2014; Le Cong et al. 2013; Ran et al. 2013). Another important improvement has been the expansion of target sites by changing the PAM requirements through engineering. Two modified SpCas9 nucleases, SpCas9-NG and xCas9, were demonstrated to target to NG PAMs in human cells (Hu et al. 2018; Nishimasu et al. 2018). Application of both enzymes has dramatically increased the numbers of potential target sites in plant genomes (Ge et al. 2019; Hua et al. 2019; Ren et al. 2019; Wang et al. 2019; Zhong et al. 2019). Thus, both, the identification of CRISPR/Cas variants and engineering of known CRISPR/Cas nucleases, have accelerated the speed of CRISPR/Cas technology development. In Table 1, we summarized different variants and orthologs of CRISPR/Cas reported in plants besides the frequently used SpCas9, SaCas9 and LbCas12a.

**Table 1:** Newly engineered CRISPR-associated proteins variants and orthologues in plants

Cas	PAM	Engineered	Property	Reference
SpCas9-HF1	NGG	N497A/R661A/Q695A/Q926A	Low efficiency	Liang et al. (2018), Zhang et al. (2018, 2017)
HypaCas9	NGG	N692A/M694A/Q695A/H698A	Low efficiency	Liang et al. (2018)
eHF1‐Cas9	NGG	N497A/R661A/Q695A/ K848A/Q926A/K1003A/R1060A	High fidelity	Liang et al. (2018)
eHypaCas9	NGG	N692A/M694A/Q695A/H698A/K848A/K1003A/R1060A	High fidelity	Liang et al. (2018)
eSpCas9 1.0	NGG	K810A/K1003A/R1060A	High fidelity	Zhang et al. (2018, 2017)
eSpCas9 1.1	NGG	K848A/K1003A/R1060A	High fidelity moderately decreased editing	Zhang et al. (2018, 2017)
xCas9 3.6	NG	E108G/S217A/A262T/S409I/E480K/E543D/M694I/E1219V	Low efficiency	Hua et al. (2019), Wang et al. (2019)
xCas9 3.7	NG	A262T/R324L/S409I/E480K/E543D/M694I/E1219V	Low efficiency	Niu et al. (2020), Veillet et al. (2020b), Ge et al. (2019), Hua et al. (2019), Wang et al. (2019), Zhong et al. (2019)
SpCas9-NG	NG	R1335V/L1111R/D1135V/G1218R/E1219F/A1322R/T1337R	Highly flexible PAM	Li et al. (2020d), Niu et al. (2020), Qin et al. (2020b), Veillet et al. (2020b), Zeng et al. (2020), Endo et al. (2019), Ge et al. (2019), Hua et al. (2019), Negishi et al. (2019), Ren et al. (2019), Zhong et al. (2019)
SpRY	NGD NAN	A61R/L1111R/D1135L/S1136W/G1218K/E1219Q/N1317R/A1322R/R1333P/R1335Q/T1337R	Highly flexible PAM	Ren et al. (2021), Xu et al. (2021)
XNG‐Cas9	NG GAN	R1335V/A262T/R324L/S409I/E480K/E543D/M694I/L1111R/D1135V/G1218R/E1219V/E1219F/A1322R/T1337R	Highly flexible PAM	Niu et al. (2020)
iSpyMacCas9	NAA	SpCas9 with the PAM interacting domain from *Streptococcus macacae* Cas9, and R221K/N394K mutations	A-rich PAM	Sretenovic et al. (2020)
ScCas9	NGA NG		Various efficiency	Wang et al. (2020c)
LbCas12a RR	TYCV CCCC	G532R/K595R	Flexible PAM	Zhong et al. (2018), Li et al. (2018c)
LbCas12a RVR	TATG	G532R/K538V/Y542R	Altered PAM	Zhong et al. (2018), Li et al. (2018c)
enLbCas12a	TTTV	D156R/G532R/K538R	Moderate efficiency	Schindele and Puchta (2020)
ttLbCas12a	TTTV	D156R	High efficiency	Huang et al. (2021), Merker et al. (2020), Schindele and Puchta (2020)
AsCas12a	TTTV		Moderate efficiency	Bernabé-Orts et al. (2019), Kim et al. (2017), Tang et al. (2017)
FnCas12a	TTV TTTV KYTV		Moderate efficiency	Zhong et al. (2018), Begemann et al. (2017), Wang et al. (2017b), Endo et al. (2016b), Liang et al. (2018)
FnCas12a RR	TYCV TCTV	N607R/K671R	Flexible PAM	Zhong et al. (2018)
FnCas12a RVR	TWTV	N607R/K613V/N617R	Flexible PAM	Zhong et al. (2018)
AacCas12b	VTTV		Efficient at high temperature	Ming et al. (2020), Wang et al. (2020d)
AaCas12b	VTTV		High efficiency	Ming et al. (2020)
BthCas12b	ATTN		Low efficiency	Ming et al. (2020)
BhCas12b v4	ATTN		Moderate efficiency	Wu et al. (2020)
BvCas12b	ATTN		Moderate efficiency	Wu et al. (2020)

From the start, plants are difficult subjects for gene editing as they have long reproductive circles and often show a low transformation efficiency. On top of this, the use of sophisticated CRISPR/Cas systems, which had been shown to work efficiently in other organisms, has led to mixed results in plants. Codon-optimization of Cas open reading frames is helpful but no guarantee for high cutting efficiencies. There are many more factors hypothesized to influence editing efficiency. One important example is temperature sensitivity: The comparison of different Cas12a variants in plants, AsCas12a, FnCas12a and LbCas12a, demonstrated that temperature is limiting editing efficiency considerably (Malzahn et al. 2019). Some loci showed drastically enhanced editing efficiencies by LbCas12a when the experiments were performed at 29 °C instead of 22 °C in Arabidopsis. Moreover, it is advisable to test multiple nuclease orthologs to identify the most efficient variants for plant genome editing. An examination of several Cas12b orthologs revealed that AaCas12b was the most efficient one in rice (Ming et al. 2020), whereas other variants, AacCas12b, BvCas12 and BhCas12b v4, were demonstrated to work well in rice, cotton and Arabidopsis (Ming et al. 2020; Wang et al. 2020d; Wu et al. 2020). Another example of mixed results is ScCas9, which was originally reported to have a minimal PAM requirement of NNG (Chatterjee et al. 2018). However, it has been shown in rice that ScCas9 works best with NAG PAMs and its mutagenesis efficiency varied drastically between different loci (Wang et al. 2020c). All these examples show that there can be a huge variation in results when newly developed CRISPR/Cas systems are transferred from other organisms to different plant species. Despite all these experimental difficulties, some CRISPR/Cas improvements were developed in plants first. Based on knowledge obtained from the work on enAsCas12a (Kleinstiver et al. 2019), an improved “temperature-tolerant” LbCas12a (ttLbCas12a) which harbors a D156R point mutation, was developed which had significantly higher editing efficiency in plants in comparison to the wild type enzyme (Huang et al. 2021; Schindele and Puchta 2020). Later on, higher editing efficiencies were also reported in human cells and fungi using this D156R mutated variant (Roux et al. 2020; Tóth et al. 2020). Another engineering strategy is the combination of mutations from different Cas variants. For example, XNG‐Cas9 which carries mutations from both xCas9 and Cas9-NG demonstrated higher editing efficiency than both parent Cas9 variants (Niu et al. 2020), whereas combining modifications of eSpCas9 1.1, SpCas9‐HF1 and HypaCas9 in eHF1‐Cas9 led to a reduction of off-side activity (Liang et al. 2018). Engineered CRISPR/Cas nuclease variants that performed well in the genome editing of plants, have—due to their DNA binding capacity—the potential to efficiently control transcription or epigenetic changes in plants, too. Modification of the guide RNA is another strategy for improving CRISPR/Cas efficiencies and capabilities. The development of the single guide RNA (sgRNA) of SpCas9, a fusion between tracrRNA and crRNA, saves time during the cloning process (Jinek et al. 2012). Further manipulation of the sgRNA provides the possibility of CRISPR/Cas-mediated targeting of different kinds of enzyme activities to specific sites in the genome. The incoporation of MS2 and other kinds of aptamers into the sgRNA for sequence-specific protein binding has been used in plants for transcriptional control, live imaging of chromosomes (Khosravi et al. 2020; Lowder et al. 2018), as well as for base editing. Multiplexing techniques require the simultaneous expression of multiple guide RNAs. This can be achieved via multiple expression cassettes of single sgRNA or multiple sgRNAs can be processed from one transcript using ribozymes. Another strategy consists of tRNA setups. One advantage of these self-processed sgRNAs or crRNAs is that they can not only be transcribed by ubiquitous expressing Pol III promoters but also by many Pol II promoters with cell-type/tissue specific expression or inducible expression (He et al. 2017). Previous reports have also shown that the efficiency of mutagenesis by a tRNA-processed sgRNA is higher than simple sgRNA expression by the same Pol III promoter (Zhang et al. 2018).

Although engineered CRISPR/Cas tools provide ample options for genome editing, potential pitfalls have to be taken into account. A relaxed PAM requirement of SpCas9 might not only result in a higher number of potential target sites, but also in a reduced activity at canonical PAMs in plants (Ge et al. 2019; Hua et al. 2019; Ren et al. 2019; Wang et al. 2019; Zhong et al. 2019). This might be due to the fact that the presence of many more putative PAM sequences in the genome might delay the correct binding to the target (Globyte et al. 2019). This hypothesis might also explain why SaCas9 with a longer PAM of NNGRRT has been more efficient at inducing mutations than SpCas9 or Cas12a in comparable experiments in plant cells (Raitskin et al. 2019; Steinert et al. 2015). Moreover, PAM relaxation might reduce the specificity, which needs to be investigated carefully. A recent report revealed that a PAM-less Cas9 might cleave the gRNA expressing cassette in the T-DNA (Qin et al. 2020b). Small Indels in the gRNA cassette will not immediately destroy the function of the sgRNA, but instead produce a mutated sgRNA, increasing the possibility of off-side targeting. This problem can be solved by changing the sgRNA scaffold sequence but such threat was taken into account when the newest developed Cas9 variants, SpG and SpRY, both with strongly reduced PAM requirement were applied (Walton et al. 2020). However, despite harboring a few potential pitfalls, the development of various CRISPR/Cas tools provides plant biologists with novel tools for genome engineering in plants. An increase in editing efficiency can be achieved by enhancing expression of the Cas9 protein, e.g. by use of a viral replicon, the inclusion of introns in the Cas9 open reading frame, the addition of a translational enhancer, or the suppression of RNA silencing (Mao et al. 2018; Peng et al. 2020; Ramona Grützner et al. 2020; Yu et al. 2020). A tissue-specific induction system allows the analysis of phenotypic consequences of a gene knockout in individual organs or cell types that would be lethal if induced in the germline (Decaestecker et al. 2019; Wang et al. 2020a).

### Advances of the base editing technique

A very important aim of using the CRISPR/Cas system is to achieve precise, predesigned genome modification. As the efficiencies of GT could not be increased to more than a few percent of the transformation events, BE is of special importance for plants. BE is based on the combination of a CRISPR/Cas DNA binding module with a nucleotide base deaminase to achieve one single or a few desired base exchanges. There are two major categories of base editors: cytosine base editors (CBE) that convert C-to-T and adenine base editors (ABE) that convert A-to-G, using a cytosine or adenosine deaminase, respectively. In most cases, nCas9 (D10A) is used in BE to generate a nick in the gRNA binding DNA strand, which enhances the efficiency of the conversion of the nicked strand (Fig. 1) (Bharat et al. 2020).

Multiple studies have shown that both, CBE and ABE, are applicable to different plant species [for reviews see: (Bharat et al. 2020; Zhang et al. 2019; Zhu et al. 2020)]. CBE experiments have been performed with high efficiency in plant cells early on. In contrast, the initial application of ABE in plants resulted in low efficiencies (Hua et al. 2018; Li et al. 2018b; Yan et al. 2018). Recently, by using an optimized adenosine deaminase (Hua et al. 2020b) and increasing PAM accessibility by SpCas9-NG in rice (Hua et al. 2019), improvements of ABE could be achieved. Although CBEs are usually more efficient than ABEs, one advantage of ABEs compared to CBEs is their low off-targeting activity, as demonstrated in the genome of rice and mouse (Jin et al. 2019; Lee et al. 2020). Via whole genome sequencing, it was found that mutations occurred more frequently in transcribed regions, suggesting that single stranded DNAs exposed by transcription are more accessible to cytidine deaminases in a non-specific manner (Jin et al. 2019). Fortunately, the problem of off-target editing of CBE could be solved by the development of two variants, A3Bctd-VHM-BE3 and A3Bctd-KKR-BE3, that have shown high specificity in rice plants at the cost of a reduction in efficiency (Jin et al. 2020).

BEs achieve precise genome editing using their narrow editing window at the target site. Although the ranges differ among various BEs, they are mostly restricted to a 10-bp region (Rees and Liu 2018). As availability of PAM sites is the major restricting factor for access to target sites in the genome, different Cas9 variants can be fused with base deaminases to expand the target range for BE. For example, Cas9-NG, ScCas9 and iSpyMacCas9 were applied to plants, using NG, NAG, and NAAR PAMs, respectively (Hua et al. 2019; Sretenovic et al. 2020; Wang et al. 2020c). Also, the development of SpRY with very low PAM restriction was applicable for BEs in plants (Ren et al. 2021; Walton et al. 2020; Xu et al. 2021). BE by Cas12a or its PAM altered variants has been successfully performed in human cells (Kleinstiver et al. 2019; Li et al. 2018a), but has not been reported in plants to date.

**Fig. 2:**
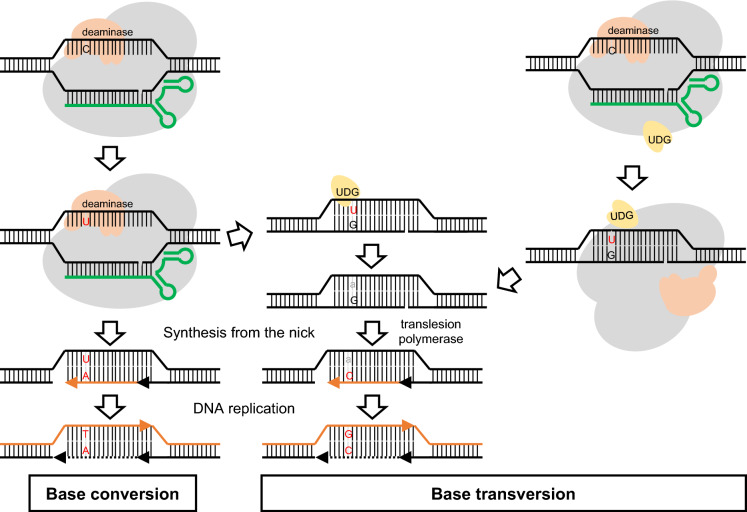
Hypothetical pathway of C-to-G base transversion by using the BE technology. After deamination of the cytidine (C, black), the resulting Uracil base (U, red) is eliminated from the DNA backbone by endogeous uracil-DNA glycosylases (UDGs) or UDG-fused base editors, resulting in an abasic site (**a**, grey). During DNA repair or replication, translesion polymerase might incorporate a C opposite to this abasic site by a template-free polymerization. Thus a C-to-G transversion is obtained

Besides direct fusions of Cas9 proteins and deaminases, base editors were engineered by using aptamer containing sgRNA scaffolds to recruit cognate binding proteins fused to deaminases, and used to generate base edited plants (Li et al. 2020c). Worth mentioning is a different kind of innovative application of ABEs or CBEs, the production of precise short deletions, as demonstrated in rice and wheat (Li et al. 2020c; Wang et al. 2020b). Another intriguing achievement is the dramatically enhanced efficiency of BE by fusion of single stranded DNA binding domain from Rad51 (Zhang et al. 2020), which increases the accessibility of the substrate to the deaminase. However, it should be tested whether this type of fusion will lead to an increase of non-specific mutations in plants. The deaminase itself is also a promising target for improvement, for example, an engineered adenine deaminase (ABE) which carries eight amino acid exchanges in respect to the wild type enzyme was able to enhance 2- to threefold higher editing efficiency in human cells (Gaudelli et al. 2020). A striking new development is a novel kind of BE that has been shown to efficiently induce C-to-G base transversion (Arbab et al. 2020; Kurt et al. 2020). Since the authors did not come up with an explanation for the mechanism, we want to suggest a scenario that is able to explain the phenomenon: after deamination of the C, the resulting U gets eliminated from the DNA by host factors, leaving behind an abasic site. During replication, a translesion polymerase might then incorporate a C opposite to this abasic site by a template-free polymerization. In the next replication cycle, this C serves as template for a G, resulting in the reported transverison (Fig. 2). Intriguingly, occasional C to G transversions were reported before from CBE studies in cotton and rice (Li et al. 2017; Qin et al. 2020a). Therefore, it will only be a matter of time until base editors for transversions will be successfully established in plants, too.

### Improvement of gene targeting

The most ancient form of precise genome editing is GT, using the endogenous HR repair machinery (Paszkowski et al. 1988). Over decades, the extremely low efficiencies of GT experiments hindered any practical applications in plants [for reviews see (Huang and Puchta 2019; Puchta and Fauser 2013)]. A solution to the problem came into reach when it could be demonstrated that site-specific DSBs can enhance GT frequencies by orders of magnitude (Puchta et al. 1996). The application of CRISPR/Cas for the induction of DSBs has become the method of choice for GT, although in most cases site-specific DSBs are repaired by NHEJ, which is the dominant DNA repair pathway in somatic plant cells. Following a number of technical advances during the last years, CRISPR/Cas induced GT could be further improved.

To obtain heritable GT evens, the mutations have to be either transferred to or directly induced in the germline. A successful strategy is the direct DSB induction in egg-cells as has been shown by two different groups in Arabidopsis (Miki et al. 2018; Wolter et al. 2018). One of the approaches consists of an all-in-one construct, including egg-cell promoter driven SaCas9, sgRNA expressing cassette and GT donor. Transformation of this construct into Arabidopsis and analysis of the T2 progeny resulted in individual lines with GT efficiencies of up to 5% (Wolter et al. 2018). Another approach is a two-step process, using sequential transformation for GT (Miki et al. 2018). Using the same egg-cell promoter, a SaCas9 construct was transformed without sgRNA and GT donor. After identifying the transgenic lines with the highest mutation induction capability out of a larger number of candidates, sgRNA cassette and the donor sequence were transformed into these selected lines after propagation to achieve GT. In this case, GT efficiencies of 5–10% could be achieved in two specific lines. The high efficiency of GT might be explained by the fact that in both cases, by involving a large number of Cas9 expressing lines, high expressers could be selected. In addition to nuclease abundance, the nature of the nuclease might help to enhance GT efficiencies. Cas12a cutting differs from Cas9 not only in that it produces 5′overhangs instead of blunt ends or sometimes one nucleotide overhangs (Stephenson et al. 2018), but that the cut is set further away from the seed sequence, which does not tolerate any mismatches during gRNA binding. In case of Cas12a, this might allow repeated cleavage, even if minor NHEJ mutations have been introduced. Several independent recent studies indicated that LbCas12a is indeed able to outperform Cas9 for GT in plants (Li et al. 2020a; van Vu et al. 2020; Wolter and Puchta 2019). GT efficiency could be further increased in Arabidopsis and tobacco using the temperature tolerant version of LbCas12a (Huang et al. 2021; Merker et al. 2020). The system used in Arabidopsis was the “*in planta* GT” approach. Here the targeting vector is transformed together with the nuclease on a T-DNA resulting in stable lines. By cleavage of target locus as well as the sites flanking the homologous region in the integrated vector, the GT reaction is induced (Fauser et al. 2012). The system was originally set up for Arabidopsis. Recently, it was demonstrated that *in planta* targeting is also applicable to corn by use of a heat shock promoter-controlled Cas9 expression during tissue culture (Barone et al. 2020). The successful induction of GT in corn may not only be due to the strategy of vector activation. Increasing the growth temperature might improve Cas9 activity as well. A different way to activate the GT vector is to increase the number of donor molecules inside cells. This has been achieved successfully in plant cells using geminiviral replicons (Baltes et al. 2014; Čermák et al. 2015, 2017; Dahan-Meir et al. 2018; Gil-Humanes et al. 2017; van Vu et al. 2020; Wang et al. 2017a). These DNA virus-derived replicons can not only be used to increase the number of GT donors but also the numbers of nuclease expressing cassettes. It has been shown that both approaches contribute individually to the enhancement of GT. The geminiviral replicon in its simplest set up consists of an expression cassette of a replication protein (Rep), in combination with the large intergenic (LIR) and small intergenic regions (SIR), forming a circular replicon. Components from wheat dwarf virus are applicable for GT in cereal cells (Gil-Humanes et al. 2017; Wang et al. 2017a) whereas bean yellow dwarf virus-derived components have been used in *Solanaceous* species (Baltes et al. 2014; Čermák et al. 2015; Dahan-Meir et al. 2018; van Vu et al. 2020). However, the application of geminiviral replicons for GT has proven to not always be successful. Till now, no report has been published on the production of fertile cereals using this technology. Moreover, efforts to use this technology for heritable GT events in Arabidopsis have failed (Hahn et al. 2018; Pater et al. 2018; Shan et al. 2018). As the Rep protein required for geminiviral replication is involved in the hijacking of the endogenous replication machinery for its DNA synthesis, transfer to the germ line as well as efficient regeneration of single cells to a fertile plant might be limited in presence of viral replicons—at least in a subset of plant species.

A major reason for the low GT efficiency is that plant cells use NHEJ as dominant DSB repair pathway rather than HR. Manipulation of the endogenous DNA repair system or expression of exogenous proteins have been used to enhance GT efficiencies in a number of previous studies. It has been demonstrated that GT efficieny can be increased in Arabidopsis or rice after factors of the classical NHEJ pathway had been knocked out (Endo et al. 2016a; Qi et al. 2013). It will be interesting to test whether suppression of NHEJ by transcriptional repression or Cas13-mediated mRNA degradation [for reviews see: (Mahas et al. 2018; Wolter and Puchta 2018)] will help to enhance GT if combined with the most promising GT approaches. Similarly, overexpression of HR-stimulating proteins could be considered as well. Overexpressing RAD54 from yeast in Arabidopsis egg cells has led to an increased GT efficiency, although no SSN was used in this experiments (Even-Faitelson et al. 2011). However, an approach using the bacterial strand exchange protein RecA did not help to improve DSB induced GT (Reiss et al. 2000).

A novel innovative approach, named tandem repeat-homology-directed repair strategy (TR-HDR), has recently been established. It uses a two-step strategy to obtain also larger predesigned genomic changes like classical GT, but differs in the involved repair pathways (Lu et al. 2020b). First, the authors achieved site-specific insertion of the donor DNA by NHEJ. The double-stranded DNA was chemically modified at both ends in both strands by two nucleotides with phosphorothioate linkages and phosphorylated 5′ ends. These modifications block the degradation of the double-stranded DNA by cellular nucleases but do not inhibit integration. An at least tenfold enhancement of site-specific integration of the extrachromosomal DNA by NHEJ into the DSB site could be achieved. To obtain a seamless predefined modification of the target locus, the authors included a sequence homologous to the target in the template in such a way that a tandem repeat with the desired mutations arose after integration at the genomic locus. In the second step, a DSB was induced by CRISPR/Cas between these tandem repeats. It has been known for a long time that a DSB induced between tandem repeats is repaired efficiently via single strand annealing (SSA) (Siebert and Puchta 2002). This kind of repair, in contrast to classical HR, is as efficient as NHEJ in somatic plant cells. It is non-conservative and leads to the loss of one repeat, including the sequence between the repeats (Puchta 2005). Thus, by combination of a more efficient site-specific integration strategy, based on modified template using NHEJ, induction of SSA and an extra investment of time, the authors were able to increase the efficiency in comparison to classical DSB induced GT which depends on the synthesis strand annealing mechanism of HR (Puchta 1998). A pitfall of this technology might be that due to the large amount of DNA supplied by particle bombardment in the first step, a lot of vector DNA is integrated ectopically at undesired sites of the same genome, which in the end might hinder the production of transgene-free mutants. In this respect, the geminiviral as well as *in planta* approaches of GT are superior to TR-HDR. In case of *in planta* GT only a single copy of the vector is available in the cells, excluding simultaneous ectopic integrations. On the other side, geminiviruses seem to have a mechanism to hinder their integration in the host genome, as this might result in virus resistant plants. Till today, integration has not been reported in case of the virus derived replicons.

### Prime editing: critical evaluation

**Fig. 3:**
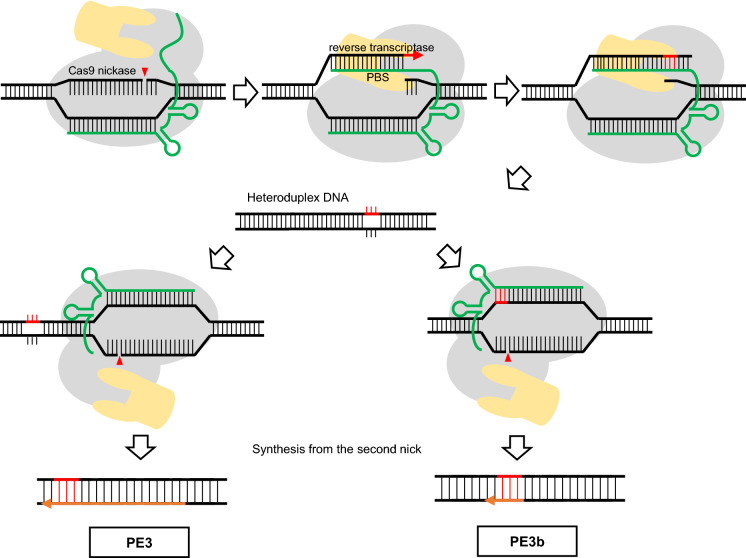
Prime editing techniques use two nicks. Prime editing (PE) uses a PE guide RNA (pegRNA) and a Cas9 nickase (H840A) fused to a reverse transcriptase to achieve precise genome modifications. After generating the first nick, the pegRNA can be used as the template for reverse transcription, using the primer binding site (PBS) paired with the target sequence. The modified sequence is incorporated only into the nicked strand, resulting in a heteroduplex DNA. A second nick is induced in the other unedited strand to ensure that the modified strand is used for mismatch repair. In PE3 approach, the nick is introduced in the unedited strand away from the first nick site. In PE3b, the second nick is induced only after the modification was incorporated to prevent deleterious outcomes by the presence of paired nicks

As all approaches to achieve precise genome modification discussed until here have their pitfalls either due to low efficiency, strong off-targeting effects, or a small editing window, hopes were flying high in the plant community when an innovative and novel genome editing technique, PE was first introduced. It allows the introduction of different kinds of genomic changes with high efficiency in mammalian cells (Anzalone et al. 2019). The technology relies on a novel CRISPR/Cas9 complex, which is composed of a PE guide RNA (pegRNA) and a protein consisting of a Cas9 nickase (H840A) fused to a reverse transcriptase. The pegRNA can be used as template for reverse transcription. It is a modified sgRNA that contains a primer binding site (PBS) and the sequence to be copied in the genome at its 3′ end (Fig. 3). This pegRNA also acts as sgRNA to define the targeting site of the nickase. The nick is induced in the non-protospacer binding strand and required for the release of a free 3′single stranded DNA end that can be used by the reverse transcriptase as a primer to copy the RNA template sequence into DNA. In this way, the designed modifications can be incorporated seamlessly into the genome. As the change of DNA sequence is limited to only one strand, it is important to safeguard the newly induced mutations from mismatch repair. Therefore, a second nick by another sgRNA is introduced in the complementary DNA strand, which deludes the cellular DNA repair machinery to preserve the sequence in the newly formed double strand in direction of the freshly induced changes. Nicks are present in the newly synthesized but not in the template strand during semiconservative DNA replication. To preserve the genetic information and eliminate mutations, the sequence of the nicked strand will generally be converted by the repair machinery. There are two different strategies: either the nick is introduced in the unedited strand away from the original nick site (called PE3) or directly opposite of the induced change by a sgRNA that only binds to the DNA sequence which had been newly synthesized by the reverse transcription (called PE3b). Thus, use of PE3b ensure that the second nick can only be induced after the first nick was removed during the repair reaction. In case of PE3, paired nicks can arise, which can result in mutagenic Indels, as has been shown in plants (Schiml et al. 2016). In case of PE3b, less unwanted Indel products were recovered than with PE3 (Anzalone et al. 2019).

A number of independent studies has demonstrated that PE is able to achieve genome modifications in plant protoplasts as well as stable lines, but with low efficiency. These studies were mostly performed in rice but also in tomato, potato and maize (Butt et al. 2020; Hua et al. 2020a; Jiang et al. 2020; Li et al. 2020b; Lin et al. 2020; Lu et al. 2020a; Tang et al. 2020; Veillet et al. 2020a; Xu et al. 2020). Obviously, a couple of different factors might influence the editing efficiency in plants, such as the nature of the reverse transcriptase, thermal conditions, length of the template, length of PBS and the requirement of the second nick. In contrast to initial reports in human cells and yeast, the generation of a second nick in the PE3 and PE3b system did not enhance editing efficiencies in plants (Butt et al. 2020; Lin et al. 2020; Tang et al. 2020). Surprisingly, a plant-specific reverse transcriptase from Cauliflower mosaic virus has shown a lower editing efficiency than a codon-optimized M-MLV (Lin et al. 2020). Editing efficiency rises with temperature: it was twofold higher at 37 °C than 26 °C (Lin et al. 2020). However, no significant differences were observed between incubation at 37 °C and 32 °C (Tang et al. 2020). Another important factor to be considered is what length the reverse transcription template needs to have, to still be copied into the genome with reasonable efficiency. Templates of sizes between 10 and 20-bp have been successfully used, but editing efficiency massively decreases with increasing template length. Major drawbacks of PE in plants are the massive variability of efficiency between loci but also the unwanted Indel production as by-product of the reaction. Depending on the locus, much more unwanted Indels than predesigned template changes were detected. Both kind of mutations were also found in combination. It is still not clear which process steps of PE in plants are the bottlenecks to further increasing editing efficiency. One possibility is that the flap structure between the DNA-RNA complex after reverse transcription might not be processed efficiently in plants. The replacement of the nickase of SpCas9 with the one of SaCas9 has proven to not be successful: only low efficiency or no PE events were observed in the respective experiments (Hua et al. 2020a). However, the result is not too surprising due to the reported low Indel activity of the SaCas9 (N580A) nickase (Friedland et al. 2015).

By now, PE has been demonstrated to work in several plant species. Various parameters might influence its efficiency. Will PE replace BE or GT for predesigned site-specific changes in plant genomes if frequencies get better in the long run? We do not think so. At this moment, PE is simply too inefficient to be considered as an alterative for inducing single base changes in plant genomes. A batch to batch comparison between PE and BE showed mostly lower efficiency of PE than BE, except for a few exceptional loci (Lin et al. 2020). Comparing GT and PE, PE is applicable to the change or insertion of a few nucleotides and GT allows us to integrate predesigned changes in the plant genomes in the range from 10 to 10,000 bp. Nevertheless, the development of PE in plants is still at its infancy. Hopefully, efficiency will rise with effort and time invested similar to what we experienced with the improvements in GT over the years. Therefore, PE, in its specific application window, has definitely the potential to become a promising tool for precise genome editing in crops.

## Chromosome engineering

**Fig. 4:**
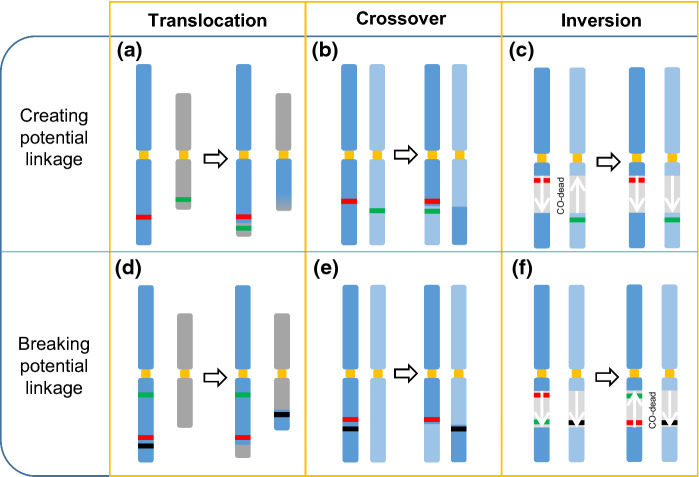
Chromosomal rearrangements and their potential application for breeding. The controlled induction of chromosomal rearrangements will enable plant breeders to change the linkage between traits (as illustrated in green, red and black). A novel linkage of two beneficial traits (in green and red) could be created by reciprocal translocations between non-allelic chromosomes (**a**) or by artificial crossovers (COs) between allelic chromosomes (**b**). Translocations can also be used to break the linkage between an elite trait (red) and an adverse trait (black) (**d**,** e**). Inversions could be used to activate or deactivate meiotic COs in a specific chromosomal region: A CO-dead inverted region could be reversed, making it possible to bring together two beneficial traits from two cultivars (**c**). The genetic linkage of two beneficial traits on the same chromosome can be fixed by inverting a region containing the respective traits, making this region inaccessible to COs (**f**)

The central aim of breeding is to combine the agronomically best traits of the gene pool of a crop species and to eliminate adverse traits from elite cultivars. Both is possible if the genes coding for the respective favorable traits are not linked to traits with detrimental features. As genes are organized in chromosomes like beads on a string and in most cases are inherited as a unit, this is often not possible, especially if gene loci are physically close to each other. Thus, breeders have a need for technologies to break or stabilize genetic linkages. Obviously, the most direct way to achieve genetic exchange between chromosomes is the induction of a crossover (CO) between homologues in the respective region. In principle, this should be achievable by the use of CRISPR/Cas (Fig. 4). In an outstanding study, the group of Avi Levy demonstrated that targeted DSBs can induce somatic HR using a homologous chromosome as template (Filler Hayut et al. 2017). They developed a selection system in tomato hybrids to identify HR between homologous chromosomes, based on a visual marker gene and single nucleotide polymorphisms. They were able to identify somatic HR events, mostly gene conversions and a putative CO event that, unfortunately, could not be transferred to the next generation. Although this study indicates that “targeted COs” via DSB-induced somatic HR can be accomplished, more work has to be invested to obtain heritable events and estimate the frequency of their occurrence.

However, also other kinds of heritable CRs can be achieved by the use of CRISPR/Cas, that might be useful for breeders. In plants, different kinds of CRs occurred not only frequently during evolution, they also can be regarded as one of the driving forces for genome evolution and speciation [for review see: (Schmidt et al. 2019b)]. Moreover, CRs are not rare events but can be observed occasionally during crop breeding or T-DNA transformation (Hu et al. 2017). Inversions of large chromosome regions of F1-hybrids are known to suppress COs (Drouaud et al. 2006; Giraut et al. 2011). Thus, by reverting inversions, genetic linkages between traits that could not be broken before, will become accessible to meiotic COs. An artificially induced inversion might be useful to stabilize a combination of beneficial traits.

Some time ago it could be demonstrated in plants that the simultaneous induction of two DSBs on one chromosome can cause not only a deletion of this region (Siebert and Puchta 2002), but with lower efficiency also its inversion (Schmidt et al. 2019a). Remarkably, a 1.1 Mb natural inversion on chromosome 4 of the Arabidopsis cultivar Col-0 could be recently reversed using SaCas9 for DSB induction (Schmidt et al. 2020). Several independent inversions could be obtained, indicating the efficiency of the used protocol. The resulting revertants were then crossed with an Arabidopsis cultivar devoid of the natural inversion. The authors were able to document COs within the formerly CO-dead region of the genome in the hybrids. Applying this method to crops should allow to restore the CO activity in known inverted regions or to prevent COs between two elite traits (Fig. 4).

In addition, induction of two DSBs on different non-allelic chromosomes can cause reciprocal translocations (Pacher et al. 2007). Whereas cells with dicentric or acentric chromosomes will be lost, reciprocal translocations that maintain the functional chromosome organization should be heritable. Indeed, heritable CRISPR/Cas9-mediated reciprocal translocations were obtained recently by the use of SaCas9 in Arabidopsis (Beying et al. 2020). The authors could induce exchanges in the Mbp range between chromosome 1 and 2, as well as 1 and 5. By cytological and molecular analysis, it could be demonstrated that in some of the translocation lines not a single nucleotide was lost during the exchanges. Thus, reciprocal translocations might turn out to be a novel way to break genetic linkages in a way that was not possible for breeders till now. Similarly, two genes on different chromosomes can be genetically linked if they are placed on the same chromosome in close proximity by an induced translocation (Fig. 4).

### Cell culture-free genome editing

**Fig. 5:**
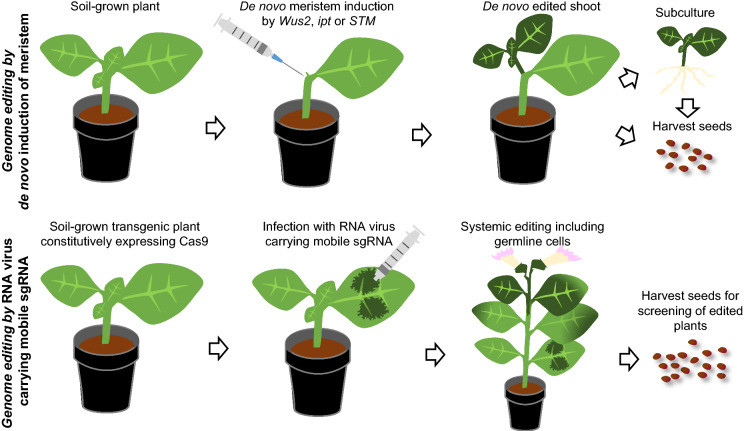
Tissue culture-free plant gene editing. Two innovative approaches to obtain gene edited plants without a tissue-culture process, as demonstrated in tobacco *Nicotiana benthamiana*. The upper scheme shows that the de novo meristem can be induced by overexpressing growth regulators, such as *Wus2*, *ipt* or *STM*. Novel shoots will be induced which will carry the desired edit in the genome. The shoots could either be propagated for regeneration or might set flowers directly, so that edited seeds should be obtained one way or the other. The lower part of the figure shows that in plants carrying a Cas9 expressing transgene, gene editing can be achieved via a systemic infection with a viral RNA replicon carrying a mobile sgRNA. There is a high probability that shoots and flowers growing after the infection are edited in their genome, resulting in edited seeds in the long run

One of the limitations of genome editing in plants is the transformation process. The three major transformation methods for plants are Agrobacterium-mediated transformation, biolistic transformation and PEG mediated transformation. Except for Arabidopsis and its close relatives amenable to floral dip transformation, the transformation of most crops requires tissue culture in order to regenerate fertile plants from somatic cells. This step is a time-consuming process and a major bottleneck for many crop plants. It is not only labor-intensive, but can also lead to genetic or epigenetic variation. Very recently, two approaches were developed by the group of Dan Voytas, both of them being able to generate genome edited crops while bypassing the tissue culture step, either by de novo induction of the meristem or by mobile gRNAs to edit meristem (Fig. 5) (Ellison et al. 2020; Maher et al. 2020).

In tissue-culture, the stimulation of cell growth usually relies on hormone containing medium. The hormones stimulate cell division and keep the resulting callus at a similar cellular stage as the meristematic cells. However, the concentrations needed vary between species and have to be optimized individually. A big step forward has been the increase of tissue-culture efficiency by overexpressing growth factors, such as *ipt* for dicots (Ebinuma et al. 1997; Smigocki and Owens 1988) or *Wus2* for monocots (Lowe et al. 2016, 2018; Nelson-Vasilchik et al. 2018). Recently, a major advance was achieved by Agrobacterium-mediated transformation of a T-DNA with three growth stimulating factors, *Wus2*, *STM* and *ipt*, while editing was induced simultaneously. Thus, genome edited shoots could be regenerated from soil-grown tobacco without a tissue-culture step (Maher et al. 2020). This method has the potential to cut down costs and time of generating gene-edited crops.

Another recent approach with the aim of bypassing tissue-culture has been the utilization of a RNA virus vector from *Tobacco rattle virus* (Ellison et al. 2020). The virus was manipulated in such a way that it produced a sgRNA fused to the mRNA of FT, a flowering factor that is known to be able to move cell-to-cell, long distances via the phloem and can even cross grafting junctions between species. Transgenic lines of tobacco plants expressing the Cas9 gene were used for virus infection. The authors were not only able to achieve gene editing in the infected leaves, they also found higher editing frequencies in the upper parts of the plants than at the initial infection. Moreover, transfer of the mutation through the germline to the next generation was documented. By using plants with a single copy of the transgene, one might be able to obtain by the segregation of gene-edited progeny that no longer contain a Cas9 expression vector. This kind of RNA-virus mediated delivery of the gRNA should, in principle, also be applicable to BE and PE approaches.

Another method for DNA-free editing of somatic plant cells is the use of the sonchus yellow net rhabdovirus as a vector to express the nuclease Cas9 as well as the gRNA (Ma et al. 2020). Application in crops requires the use of RNA viruses which are able to carry the several kbs of excess genetic information, however the virus in question has only a narrow host range. Unfortunately, most other RNA viruses are not able to carry extra information for coding for such a large protein as Cas9. Nevertheless, as demonstrated by the recent characterization of CasΦ (Pausch et al. 2020), CRISPR/Cas nucleases of much smaller size are becoming available for editing, indicating that this approach might have a promising future as well. Moreover, it might be useful to consider further approaches such as grafting for DNA-free editing. Thus, the next years might see a growing number of options for DNA-free genome editing of plants.

## Conclusion

Over the last two years we have seen tremendous progress in the development of CRISPR/Cas-mediated genome editing tools in plants. The establishment of various natural and engineered nucleases has enabled us to target almost any sequence in the genome with high efficiency. We are now able to induce genomic changes from a single base pair to Mbps using technologies from base editing to chromosome engineering. We also have tools at our hands to overcome transformation hurdles in crops. One does not have to be a prophet to predict that also in the next years we will see further major improvements of our genome editing toolbox. This will enable us to supply breeders with the tools they need to breed crops that need less pesticide and are more tolerant to environmental stresses due to global warming. However, challenges remain, especially those beyond technological developments: A scientific based regulation of gene edited organisms everywhere around the globe, an international consensus on how to trade genome edited crops as well as a positive assessment of the technology by the general public will need to be achieved so that mankind will benefit best from these new technologies.

## Data Availability

Not applicable.
